# Coaching as a Buffer for Organisational Change

**DOI:** 10.3389/fpsyg.2022.841804

**Published:** 2022-05-31

**Authors:** Mirosława Huflejt-Łukasik, Jan Jędrzejczyk, Piotr Podlaś

**Affiliations:** Faculty of Psychology, University of Warsaw, Warsaw, Poland

**Keywords:** organizational change, coaching, objective change, subjective change, resistance

## Abstract

When introducing changes to an organisation, it is crucial to know how a given change will affect the company’s success. It is easy to forget or, more frequently, fail to appreciate the importance of the feelings and thoughts of the people who experience such changes. The distinction between objective change and subjective change is helpful in understanding the psychological consequences of changes and how they may affect the effectiveness of introducing changes in organisations. Results of studies on the psychological costs of changes for an individual indicate that there are differences in the way people experience objective and subjective changes, and that the way a change is perceived by an individual (i.e., subjective change) is crucial for the consequences of change. Studies have also identified factors which can buffer the negative consequences that changes may have on an individual. For changes in an organisation, coaching is one method to nurture these buffering factors in affected individuals, and, most of all, in those who are responsible for planning and introducing the changes, so that the employees of a company can experience the change in the most constructive way possible.

## Introduction

The rapidly changing environment of today’s globalised world makes constant adjustment of organisations necessary. Hence, every organisation must be prepared for different types of changes—in strategy, structure, adjustment to external conditions, etc—in order to meet the demands of the environment. For years, introducing changes has been considered the norm, not an exception (Hammer and Champy, 1993). This is why the topic of changes in organisations is of interest not only to practitioners but also researchers.

Despite the existence of many theories and methods regarding efficient implementation of changes in organisations (Al-Haddad and Kotnour, 2015), research consistently shows that, in most cases, changes do not bring the desired results (Beer and Nohria, 2000; Smith, 2002, 2003; Isern and Pung, 2007; McKinsey and Company, 2008; Rouse, 2011; Jansson, 2013). At the same time, increasing attention is being paid in the literature not to the content of changes *per se* (i.e., what is supposed to change or different models of implementation), but to those undergoing the changes—the employees (Armenakis and Bedeian, 1999; Erwin and Garman, 2010; Oreg et al., 2011). Some authors stress that employees’ reactions may be central to the success of an organisational change (Oreg et al., 2011).

The aim of this article is to combine the two paradigms of looking at change. On the one hand, change as the experience of an individual has for some time been the subject of research, especially in cognitive and personality psychology, from which perspective it is an internal process taking place within the self-regulation of the functioning of the individual, with its specificity on the cognitive and emotional level (subjective change) (Keyes and Ryff, 2000). Therefore, our first research question refers to not only change in organisation, but to a broader perspective: how people experience change in their life, what influences attitude to change? On the other hand, change is the subject of research from the perspective of management and organisational psychology, focusing on the spectrum of factors influencing changes implemented in organisations, companies (objective change) (Oreg et al., 2011). The second research question refers to change in organisational context: what factors are crucial in individual resistance to change implemented by organisation? A closer look at this connection between two paradigms of looking at change - in our opinion - allows us to draw conclusions for the practice, i.e., to point to the already existing, although requiring research, applications of coaching in supporting the planning and course of changes in organisations. Based on previous considerations about change we formulate the third research question: how can coaching help in implementing change in organisation?

The subsequent part of this manuscript will discuss changes from an individual’s perspective in the context of research on objective and subjective change. This area deals with the perception of any change in a person’s life, and thus also their professional life. This will allow us to focus on those factors of change which are universal, independent of individual differences or the conditions of change in a given organisation.

## Objective Change and Subjective Change From the Perspective of an Individual

Life events influence the functioning of an individual and their wellbeing (Keyes, 2000; Keyes and Ryff, 2000). When considering change from an individual perspective, it is important to recall the distinction between objective and subjective change (Huflejt-Łukasik, 2010). The former concerns observable events in one’s life—a promotion at work or loss thereof constitutes an objective change. Subjective change concerns one’s perceptions and assessments regarding objective changes. Specifically, subjective change refers to an individual’s perception of: (a) did change occur (small differences in assigned tasks may not be perceived by as change); (b) was the change big or small; (c) was the change positive or negative—e.g., a promotion may be considered to be both a positive and a negative change.

One element connecting all change is the impact on the health and wellbeing of the individual who experiences change. When experiencing an objectively negative change (e.g., loss of a job), individuals exhibit higher levels of psychopathological symptoms than those not subject to such change. Individuals who experience objectively positive change (e.g., getting their first job) also bear some psychological burdens, which can manifest somatically (Huflejt-Łukasik, 2010). Research shows (Keyes and Ryff, 2000) that every change observed by an individual, independently of its subjective assessment as positive or negative, bears some negative emotional consequences. A decrease in positive emotions and increase in negative emotions was found in individuals who experienced improvement or decline in their functioning in social roles in comparison to individuals who perceived their state as unchanged (Keyes, 2000; Keyes and Ryff, 2000). Studies of perceived, subjective change have also shown that it can incur the following costs: somatic illness (common cold, flu, etc.), increase in depressive symptoms, volatility in one’s self-image, a focus on how others see us, or an intensification of paranoid thinking (Brown and McGill, 1989; Huflejt-Łukasik, 2010).

There are differences in the experiences of changes that are perceived as negative and those that are perceived as positive. The former leads to a lower state of wellbeing, self-acceptance, and an increase in pathological symptoms. In the case of changes perceived as positive, an increase in dysphoric symptoms is observed alongside lowered self-acceptance, but also an increase in wellbeing in the self-development dimension and unchanged levels of overall wellbeing in comparison to people who do not experience change (Keyes and Ryff, 2000). Individuals who saw change for the worse also reported worse functioning, while individuals who saw change for the better reported an improvement in functioning. At the same time, individuals who reported positive change reported higher levels of psychological wellbeing in the dimension of personal development in comparison to those who remained unchanged. Both of the above groups were characterised by similar levels of satisfaction with life (Brown and McGill, 1989; Keyes, 2000; Keyes and Ryff, 2000).

Differences in the consequences of positive and negative change stem from the existence of two needs: constancy and development. The constancy, continuity, and consistency of one’s self-image ensures self-understanding and that one is governed by one’s own behaviours, which gives a sense of predictability to the world, and, in turn, is important for psychological wellbeing. An individual seeks information that is consistent with what they think about themselves and how they perceive their situation. This allows one to retain one’s self-image and the associated emotions. Self-development is associated with becoming better, strengthening one’s sense of self-worth, and maintaining a positive mood. People seek information which shows that they are developing or that they are better than others in some particular area (Brown and McGill, 1989; Swann et al., 1989; Keyes and Ryff, 2000; Huflejt-Łukasik, 2010).

Emotional and cognitive reactions to change may differ because of the aforementioned needs. The standards of consistency and self-development are impinged upon by positive and negative change in two different ways. Positive change unsettles the *status quo* and thus negatively affects the consistency of the self. At the same time, it realises the need for development, results, and achieving goals and thus it positively influences the self-development standard. Negative change impinges on both standards by interfering with the current image of self and not realising the standard of self-development (Swann et al., 1989). Referring to the example from earlier, a promotion may constitute interference with the consistency standard—i.e., somehow one is no longer a specialist but a manager, who has to adjust oneself to a new role. However, this also brings an opportunity for development. On the other hand, dismissal from work not only destroys the *status quo*, but may also drastically limit one’s chances for development and realisation of professional goals.

The novel concept of “derailment,” proposed by Burrow et al. (2018), highlights the importance of subjective perceptions of change. Its creators stress that perception of one’s identity as stable or as frequently changing is crucial to one’s wellbeing. Subjective change may lead to a sense of lack of continuity (“derailment”) between what one used to be and what one is right now (regardless of whether this is a positive or negative change). This state is associated with depressive symptoms. Interestingly, this sense is independent of the objective changes occuring in one’s life.

Self-regulation processes initiated in response to changes or threats to the self play an important role in coping with change (Huflejt-Łukasik, 2010, 2020). Their primary role is to return a sense of balance and comfort to the organism (Carver and Scheier, 1998; Carver, 2004). In this context, the process of self-focus, i.e., paying attention to oneself and not the external environment, is especially important. This serves to compare one’s current state with the desired state—i.e., the state which should occur after the change. If the goal is reached, the individual experiences relief and satisfaction; when the standard is not fulfilled, the individual experiences discomfort, which motivates them to change their actions in order to finally reach the goal (Carver and Scheier, 1998; Carver, 2004).

Brown and McGill (1989) talk about the crucial role of sense of control as a factor which minimises the consequences of the experienced change. Their research indicates that the cognitive, emotional, and motivational consequences of stressful events may be perceived as much smaller if one feels that one can influence the adverse situation. The relationship between self-esteem and influence over changes in one’s health is also important in this context. Research by Brown and McGill (1989) suggests that changes of a positive character are associated with a decline in the health of people with low self-esteem, which does not occur in the case of individuals with high self-esteem. Interestingly, the groups do not differ in this regard when negative changes occur. The consistency of the self may play a crucial role in explaining this discrepancy. Swann et al. (1989) indicates that individuals characterised by low self-consistency are more prone to negative effects of positive change. This is because of impingement on the self-standard regarding the constancy of the *status quo*. In the case of people with high self-consistency, which usually characterises people of high self-esteem, a positive change does not influence self-consistency as much. The crucial role of self-esteem in coping with stressful situations is also stressed by Roth et al. (2012). They believe that the damage to self-esteem caused by a traumatic event may lead to the development of post-traumatic stress syndrome.

The literature indicates the important role of relationships with other people for buffering the effects of stressful events (Cobb, 1976; Cohen and Wills, 1985). It is believed that interactions with others provide positive reinforcement and feedback, which leads to an increase in stability and sense of predictability. Research by Millar et al. (1988) revealed the importance of relations with other people in the context of changes. Changes interfering with an individual’s previous activities (going to college) were associated with negative consequences. The more the previous routine was interfered with, the stronger were the depressive symptoms. However, the negative effects were significantly diminished if an individual managed to find someone in their new environment with whom it was possible to share time and take part in favourite activities together. This is in-line with the research on organisational change. A review by Stouten et al. (2018) discusses the positive influence that social bonds and mutual trust among employees have on organisational change.

Summing up, the perception of change by an individual, i.e., subjective change, is more important for the psychological consequences of change. Every change, including organisational change, is associated with psychological costs and thus it is a challenge for both those who experience it and those who introduce it (who, indeed, also experience it). Two factors can buffer the negative consequences of change. First, positive beliefs about the change, which do not allow for negative assessment of things associated with the change. One’s way of thinking nullifies the negative effects of change if the change is assessed as positive. Second, meaningful interactions with other people, such as spending time together and sharing experiences and interests, are important for buffering the negative effects of change. Coaching is one method which can facilitate both of these buffering factors when an organisation is undergoing change.

The next part of this manuscript will focus on changes in organisational context. The phenomena of resistance to change and factors which affect resistance will be discussed. Later, one will find the part regarding organisational change and coaching as a helpful method.

## Changes in Organisation

The phenomenon of employees resisting change is often described as the main factor which causes organisational changes to fail (O’Connor, 1993; Vakola and Nikolaou, 2005). However, this is a natural reaction to change: going from a known situation into a new, unknown situation (Bovey and Hede, 2001). Resistance to change has been considered from many perspectives: as something that resembles cancer, which kills all progress; as a natural reaction to change which requires discussion and working-through; and as a process of little significance, which cannot be managed anyway (Palmer and Dunford, 2008).

Bareil (2013) proposed two paradigms that differently interpret resistance to change. In traditional paradigm resistance to organisational change is seen as opposing change, behaving in order to slow or stop the change. In this perspective change is planned in advance and not open to discussion: the leader of change has decided change is inevitable and others have to follow. Employees who resist have a dispositional inclination to resistance. In modern paradigm behaviours like doubting, arguing are interpreted not only as trying to stop the change, but as trying to understand and adapt to the change. In this perspective resistance is seen as a reflective reaction, form of feedback or even a resource. Leader of change can be seen also a resource and is open to discussion: change initiative may be constantly improving. Therefore, resistance can be interpreted as negative or positive phenomena. Interviews with coaches working with employees during organisation change show that not only negative emotions can be indicators of resistance, but also positive. Moreover, resistance sometimes can be seen as neutral (Brandes, 2020). Seeing change as a threat or as a resource leads to different strategies of dealing with change.

Nowadays most researchers use Piderit’s conceptualisation (Piderit, 2000), which, drawing on social psychology, defines the phenomenon as multifaceted. It consists of behavioural, cognitive, and affective components. The behavioural component includes behaviours which either foster or reject change—usually manifested as passiveness (e.g., performing one’s duties at a minimal level), but also lack of engagement in support for the change or open sabotage (Erwin and Garman, 2010). The cognitive component refers to understanding the reason behind the change and evaluating its value (both for oneself as an individual and for the entire organisation). The affective component refers to the emotions associated with change—research focuses mostly on negative states, such as anxiety, stress, tiredness, though some work also assesses positive states (e.g., satisfaction or sense of pleasure; Rafferty and Griffin, 2006; Oreg et al., 2011). Research confirms that indicators of resistance can be observed at all three components (Brandes, 2020). Because these components do not necessarily need to oppose change, Piderit (2000) emphasised that this phenomenon should be thought of as a reaction to change, rather than resistance. It is worth stressing that an employee’s reactions need not be consistent across all components—i.e., an individual may experience stress associated with change (affective component) but act in a way that supports the change (behavioural component).

Some authors posit that affective component is the most important: emotions play decisive role in attitude to change (George and Jones, 2001; Liu and Perrewe, 2005; Helpap and Bekmeier-Feuerhahn, 2016; Brandes, 2020). Emotions were not considered as an important factor in implementing changes in organisations for a long time. However, major changes are relevant for employees, therefore they lead to emotional reactions, from negative (anger, anxiety) to positive (hope, satisfaction) (Helpap and Bekmeier-Feuerhahn, 2016). George and Jones (2001) suggest changes cause discrepancy in individual schemas – cognitive structures of one’s knowledge. People are used to the structure of the organisation, ways of communication with co-workers and management, behaviours enacted in the average day of work etc. When some or most of them are changed, one has to construct new schemas, i.e., new ways of behaving or interpreting situations. Developing new schemas is usually accompanied by high cognitive effort and this process is influenced by emotions (Helpap and Bekmeier-Feuerhahn, 2016). Interpretation of new situations can be parallel to simultaneous emotions: negative while feeling negative emotions and positive during positive emotions. This in turn can lead to differences in change commitment, expectations and change efficacy (Helpap and Bekmeier-Feuerhahn, 2016). For example, while feeling negative emotions, an individual may not believe that change will have positive consequences and that he/she has abilities to implement the change, and will not commit to the change, but resist it. Emotions at the beginning of change are critical, because they are intense and will affect subsequent stages of changes and its interpretations (Liu and Perrewe, 2005). However, they are usually mixed (e.g., fear and excitement) and highly malleable.

George and Jones (2001) in their process model of individual change also posit that emotions play a central role in the change process and are a trigger that begins individual change. Emotions signal discrepancy with individual schema due to organisational change. From the perspective of an agent of change the best outcome would be if individual construct new schemas, thus adapt to change. However, George and Jones (2001) points out that dealing with discrepancy is a complex process with several stages and at each stage resistance may occur. For example, an individual instead of constructing a new schema can deal with discrepancy by denying the discrepancy, lowering its priority or interpreting discrepancy as unsolvable.

These considerations emphasise the role of cognitive and affective processes in individual changes. Emotions experienced by employees were ignored or interpreted as irrational resistance for a long time. Contemporary approaches to organisational change view emotions as a critical factor in implementing changes.

Since employees’ reactions to a change are key to its success, it is worth investigating the factors which influence such reactions. Oreg et al. (2011) proposed a complex model based on a review of 60 years of literature regarding reactions to organisational change. This model includes the precursors of reaction to change, the reactions themselves, and the consequences of change. Factors which influence how employees react to change that are relevant to the current paper will be discussed subsequently.

The expected consequences of change are one of the basic factors affecting reactions to change. An employee assesses whether they will lose or gain from the change. Many dimensions of profits and losses may be taken into account (such as prestige, safety at work, social relations, requisite skills; Cunningham et al., 2002; Giangreco and Peccei, 2005; Chreim, 2006; Oreg, 2006). The overall conclusion from the research is that the stronger the expectation of profit from change, the more frequently employees express attitudes that are supportive of the change (or less intense resistance), and that when changes are perceived as unfavourable, employees exhibit strong resistance. Also, former experiences can play a vital role (Brandes, 2020).

The relations in an organisation are another factor which has a significant impact on how change is perceived. Lack of confidence in managers leads to cynicism—loss of faith in the leaders’ ability to effectively implement change (Reichers et al., 1997). This, in turn, has a destructive influence on the employees’ motivation, engagement, and work satisfaction (Armenakis and Bedeian, 1999). Oreg (2006) indicates that lack of confidence negatively impacts all three of the components of reaction to change. The way a change is introduced also determines the way it is received. Informing employees about the nature of the change and its consequences reduces employees’ resistance and increases their openness to change (Wanberg and Banas, 2000; Lewis, 2006). Significant reduction of resistance can also be achieved by engaging the employees in the process of change (Armenakis and Bedeian, 1999; Bartunek et al., 2006). Employees’ readiness for change is another important factor. Motivational factors which may influence this readiness can be external (salary, promotion) or internal (satisfaction from work). Results of studies by Shah et al. (2017) indicate that external motivation may be an important factor at the beginning of the process of change, but long-term engagement in that process may require internal motivation.

Another factor which influences reaction to change is individual differences. Individual differences relevant to this paper include sense of self-efficacy (i.e., an individual’s belief that they have the resources to complete a given task; Wanberg and Banas, 2000; Cunningham et al., 2002) and coping styles (i.e., strategies for dealing with challenges; Judge et al., 1999; Cunningham et al., 2002). For instance, Mäkikangas et al. (2019) have shown that motivational wellbeing associated with work and core beliefs about oneself are important factors associated with a positive attitude toward organisational changes.

In the subsequent chapter of the manuscript definitions of coaching and coaching possible role in dealing with resistance and implementing change in organisation will be discussed.

## Organisational Change From the Perspective of Consulting and Coaching

Every firm must ensure that its structure and function are as flexible as its environment is changeable. A firm’s success or failure may hinge on its implementation of changes. This requires an organisation to have knowledge and skills in the areas of designing, communicating, and implementing changes in a way that is amenable to people and effective for the organisation (Penc, 1997, 2001). According to systems theory, an organisation as a social system strives for homeostasis and ‘‘reacts defensively’’ to changes introduced, i.e., it has a tendency to return to the system’s previous rules of functioning (negative vs. positive feedback loop^1^; see Senge, 1990). Regardless of the fact that classical systems theory does not explain the entire complexity of changes in systems, and thus also of changes in organisations (see e.g., Caldwell, 2012), it allows us to see phenomena such as the manifestation of resistance in the process of change as natural and necessary take into consideration at the planning stage.

In order for the process of organisational change to go as smoothly as possible, the following basic elements are necessary:

1. Awareness of the influence and meaning of change, not only for the organisation and the business realised by it, but also for employees. Managers of different ranks play an important role here.
2. Planned implementation of changes: risk analysis which takes into account the fact that the change occurs in a social environment; analysis of objections and profits from different perspectives (such as the goals of the organisation, new activities that are in-line with the market’s demands, the structure of the organisation, and employees—especially those who can influence the implementation of change). The change to be introduced is important, but so too is how it will be introduced.
3. A plan for effective implementation of changes.
4. A plan for communicating changes and effective communication of changes.
5. Implementing changes and verifying the effectiveness of this process (see Penc, 1997).

When introducing changes in an organisation, it is of particular importance to communicate them at different levels of the organisation. Awareness of changes, their inevitability, and knowledge about the actual situation of the company increases the motivation and engagement of employees in the realisation of constructive ideas. It is important to remember to communicate changes in a way that illustrates the desired and predicted results, so that the employees perceive the change as positive. Studies show that changes are more likely to fail if they are poorly communicated, when there is no agreement with employees about the goals in the context of implementation of changes, or when managers do not control these goals (Penc, 1997; see also: Armenakis and Bedeian, 1999; Erwin and Garman, 2010). Management style is also important, especially in the initial phases of introducing changes, as it is associated with the employees’ subjective assessment of changes. Research shows that a transformational style (identifying with a common shared vision, building pride and faith in employees, inspiring them, and paying attention to individual needs) is associated with positive assessment of changes, while a transactional style (rewarding employees for the realisation of clearly defined objectives) is associated with a negative assessment of changes (Holten and Brenner, 2015).

From the perspective of coaching and consulting, the most common difficulties in implementing organisational changes are: planning change without paying enough attention to its reception by employees, lack of adequate communication, and underrating the power of negative emotions and fear in the employees who are undergoing the changes. The latter involve all of the employees, but are particularly important for managers and those responsible for implementing changes at all levels in an organisation. Managers often experience a strong fear of the employees’ reception of change, their negative emotions and reactions, and fears they might express. At the same time, managers on different levels are also undergoing change and are responsible for communicating the change to their team and for its implementation. It is possible for the managers themselves to have doubts and fears or to not understand the reason behind the changes. This may make it more difficult to implement the change to the everyday activities in an organisation. Additional misunderstandings and negative emotions may also stem from the relations in organisation and the existence of hierarchy. Hierarchy is often a barrier in communicating and openly discussing change, especially if there are difficulties in the implementation process. The flow of information from the bottom to the top of the organisation, which can be particularly useful to the managers implementing change, is especially prone to blockage. Thus, change requires the mobilisation of employees, creates particular challenges, and necessitates the use of the potential and knowledge of the employees while, and at the same time, makes it particularly difficult to effectively do so. Negative emotions, which occur in the process of change, are an important obstacle in this way. Research by Rafferty and Jimmieson (2016) indicates that emotional reactions in the initial phases of organisational change may influence the employees’ wellbeing. Interestingly, this study also found that initial affective resistance was negatively related to subsequent cognitive resistance. The authors interpreted this result as suggesting that negative emotions at the beginning of the process may lead to venting these emotions or seeking social support. It is thus important to facilitate activities within an organisation that could improve the emotions of employees of all levels. This could lead to a subsequent decrease in resistance.

### Coaching as a Method

Coaching is one tool which can minimise the natural costs of changes, modify perceptions thereof, and decrease the subjective costs. It is a good tool for supporting organisational changes, and coaching for managers is of particular importance (Stober, 2008; Grant, 2014). Coaching aims to support the person receiving it in the realisation of their goals and to set in motion the necessary psychological resources and creative problem-solving (Huflejt-Łukasik and Turkowski, 2011). Different definitions of coaching can put the emphasis differently, the definition below reflects the key tasks of coaching seen as “the process of helping people and teams to perform their tasks in the most effective way possible. It includes bringing out people’s strengths, helping them to overcome internal barriers and limitations in order to achieve personal perfection.” (Dilts, 2006, p. XX). Noteworthy, especially in the context of the subject of this article, is the concise definition of coaching in the work of Joseph O’Connor and Lages, who present it as “methodology for change” (O’Connor and Lages, 2009, p. 2). Also worth noting is the definition of coaching that refers to scientific knowledge in the field of cognitive and personality psychology, and specifically to the crowning concept of self-regulation by Carver and Scheier (1998). Authors perceive coaching as a collaborative relationship formed between coach and coachee for the purpose of attaining professional or personal development outcomes which are valued by the coachee. Goals are set in order to stretch and develop an individual’s current capacity or performance. “In essence the coaching process facilitates goal attainment by helping individuals to (i) identify desired outcomes, (ii) establish specific goals, (iii) enhance motivation by identifying strengths and building self-efficacy, (iv) identify resources and formulate specific action plans, (v) monitor and evaluate progress toward goals, and (vi) modify action plans based on feedback. The “monitor-evaluate-modification” steps of this process constitute a simple cycle of self-regulated behaviour.” (Grant et al., 2010, p. 3–4). In team coaching, the definitions emphasise the process that allows to collectively create a new quality of communication, action and collaboration. Coaching is really important as “helping the team improve performance, and the processes by which performance is achieved, through reflection and dialogue.” (Clutterbuck, 2007, p. 77.) The specific nature of team coaching is emphasised: “Enabling a team to function at more than the sum of its parts, by clarifying its mission and improving its external and internal relationships. It is different therefore from coaching team leaders on how to lead their teams, or coaching individuals in a group setting,” (Hawkins and Smith, 2006).

As individual formulations of the definition of coaching existing in the literature emphasise its various aspects, it is worth noting which elements are key and characteristic of coaching, and also allow to distinguish it from other forms of supporting the development of people and organisations (such as psychotherapy, consulting, training). In the case of coaching, it is particularly important to focus on the client’s goal - its definition and implementation in the course of work, as well as the fact that the client generates a solution for his problems (O’Connor and Lages, 2009; Huflejt-Łukasik and Turkowski, 2011). Coaching is used to activate the client’s creativity and strengthen his resources so that he can achieve the results (goals) he desires. Coaching is also characterised by a symmetrical partnership relationship between the coach and the client (Huflejt-Łukasik and Turkowski, 2011).

Coaching is assumed to have a short-term formula (on average, the entire work includes several meetings), is based on challenges related to the present and the future, as well as allows flexibility in techniques and work organisation. Professionally prepared coaching is based on a contract agreed with the client and requires the coach to adhere to a number of ethical principles, including the obligation to maintain professional secrecy (Huflejt-Łukasik, 2010a,b). The coaching client may be an individual or a team. In the case of the latter, the primary goal of coaching is the common goal of the team, and the sub-goals included in the agenda of the meetings result from interviews with coaching sponsors (representatives of the organisation) and members of a given team. The starting point and the basis for coaching in organisations are the goals of the coaching project, that is directly or indirectly business goals in a given company, together with the indicators of their achievement, agreed with the representatives of the organisation (leaders, HR). For this reason, coaching in the organisation is a tool of a consultant who, on the basis of data obtained from the organisation, selects the right way to achieve the organisation’s goal, focusing on the impact on human resources - leaders, employees. Specific goals of a coaching client (coachee), established with him/her/team, should be in conformity with a coaching project’s goals that are the business company’s targets.

### Links Between Coaching Approaches and Organizational Change

At the general level, the impact of coaching on the organisation, including change in the organisation, is based on two pillars: established business goals and the quality of leadership.

Coaching affects human resources, and therefore can be a helpful support tool in those business goals where the quality of the company’s employees is important. Especially when specific challenges (e.g., planning and implementing changes) or problems (e.g., low work efficiency, conflicts in teams) are noticed and identified. The dynamically changing environment and high competition on the market force organisations to constantly change and use effective development methods (Rosha and Lace, 2016). And one of the most important factors determining whether a given organisation will be successful in the market is human capital (Zelga, 2017). Hence the popularity of coaching in the business environment (Rosha and Lace, 2016), both provided as a tool for the influence of external experts, consultants, and in the form of a coaching style of management (Huflejt-Łukasik et al., 2014). At the core of coaching competencies are the improved competences of the so-called micro leadership, allowing you to lead a team of people more effectively in the context of a given task, better seeing and understanding their individual perspectives. For formal macro leadership in organisations to be real, leaders should demonstrate micro leadership competencies (Nicholls, 1988; Huflejt-Łukasik et al., 2014). Coaching as a method at the base level leaves more space for people’s activity and ideas, strengthens their creativity and access to strengths (Huflejt-Łukasik et al., 2014). One of the translation mechanisms explaining links between coaching approaches and organisational change is also the acting upon employees motivation so as to support their internal autonomic motivation. Research revealed that when managers became more autonomy supportive it had a positive effect for their employees who reported greater job satisfaction and expressed greater trust in the top corporate management (Deci et al., 1989). They developed more positive and trusting attitudes toward the top management who would have been many levels above these employees in the organisational hierarchy and with whom these employees would not have had any contact.

One of its types of coaching, which companies often use, is business coaching (executive coaching), one dedicated to management and managerial staff (Zelga, 2017). It is focused on the development of business efficiency, improvement of management processes and activities on business goals and results. Maybe, among others, provide support in creating or checking the direction of development for the organisation, its vision, and in planning and implementing strategic goals. It is helpful in introducing organisational changes and caring for human resources (Grant et al., 2009, 2017; Grant, 2014; Grover and Furnham, 2016). Another very common one is professional coaching, which enables, *inter alia*, the development of an employee in a given professional role, as well as ongoing support in difficult and demanding situations. Most often it is offered to managers or leaders, constituting the basic method of their development in a professional role in some organisations (Jones et al., 2015; Grover and Furnham, 2016). Organisations also use team coaching, offered e.g., to members of management or project teams, i.e., formally or task-based teams (Carter and Hawkins, 2013; Huflejt-Łukasik et al., 2017; Zelga, 2017). The most common topics of team coaching are: team building, or improving the effectiveness of their work and cooperation in a team, conflict resolution, support in introducing changes in organisations, or strategic meetings to review and plan the implementation of current team goals. A specific form of coaching in an organisation is also job coaching. It consists in accompanying a person or a team in the implementation of current professional tasks, so that these direct observations can be translated into feedback and work during the session.

There are many different forms of coaching and different types of people who can benefit from coaching; however, some are particularly important for the maximally efficient introduction of changes:

1. Support in the form of individual coaching for a person responsible for planning and implementing change is key for effective implementation. This is not only an opportunity to verify the plan for change and to look at it from different perspectives, including what elements should be included and how it can be communicated to employees so as to buffer the negative effects of change. Coaching is also an opportunity for the person responsible for implementing changes in a firm to take care of their emotions during such a responsibility-laden and psychologically burdening task.
2. Team coaching is an opportunity for important information to be communicated to employees by those planning and implementing change. It is also an opportunity for bottom-up communication about perceptions and fears of change or factors important for that change from the perspective of employees. In this context, the employees may, at least partially, become the creators of change, taking some responsibility for it through their own engagement.
3. Team coaching as a support for implementing change on different levels of the organisation allows the managers at different levels to prepare for new activities and to decrease negative emotions in order to communicate the change to their teams and implement it in the best way possible. Different threads and competences may be developed depending on the needs of the firm; however, elements such as taking care of constructive emotions, inculcating positive perceptions of change, and team managers providing good solutions should take place in every such coaching meeting.
4. Individual coaching for the managers implementing the change allows them to work on their individual goals in the role of manager, and, in the context of change, it is particularly important to work on their emotions, cope with stress, and the ability to be assertive. It is also important to take into account concrete aspects of implementations and projects, and to work on team motivation and problem solving.

Studies on coaching for organisations have confirmed its effectiveness. Jones et al. (2015) conducted a meta-analysis of 17 studies on coaching in the workplace. The use of coaching brought positive results in terms of affective changes, changes regarding skills, and changes regarding levels of productivity and achieving goals. The effect size found by the meta-analysis is similar to results of other interventions used in organisations (e.g., training for managers).

At the same time, there is still little research on the effectiveness of coaching on organisational change itself (Grant et al., 2009), however the results of a study regarding individual coaching for managers undertaken in the process of organisational change could serve as an example (Grant, 2014). From the firm’s point of view, the goal of the coaching project was to ensure that managers effectively reached their goals, regardless of the turbulence associated with the organisational change. A total of 38 managers took part in the study. Assessments were conducted twice: before and after coaching. The results showed that taking part in coaching was associated with better realisation of goals, an increase in solution-oriented thinking, better coping in situations of uncertainty and change, an increase in the sense of self-efficacy and resilience in the role of manager, as well as a decrease in depressive symptoms. The levels of anxiety, stress, and satisfaction with work did not change. The authors also emphasised that the impact of coaching generalised to family life.

Research on coaching leaders (individuals responsible for the implementation of change) may also serve as indirect proof of the utility of coaching during organisational change. Grover and Furnham (2016) conducted a systematic review of research on the effectiveness of coaching in an organisation. One of the most investigated effects of coaching was its influence on leaders’ behaviours, assessed from various perspectives. Most of the studies mentioned in the review indicated that coaching had a positive influence on leaders’ behaviours. Effects on the subordinates of managers who underwent coaching were also observed: a decrease in turnover of staff and an increase in work satisfaction, engagement, and commitment to the organisation (Grover and Furnham, 2016). In one recently published study (Grant et al., 2017), the influence of coaching on individuals responsible for implementing strategic changes in the health sectors was assessed. Undergoing coaching was associated with positive effects such as better achievement of goals, higher tolerance of uncertainty, higher levels of resilience, a greater sense of self-efficacy in the context of leadership, and lower levels of stress and anxiety. The influence of coaching on these areas may play an important role during the implementation of changes in an organisation, especially taking into account that changes in the behaviour of leaders influences the subordinates’ attitudes toward the organisation.

Coaching may significantly support the implementation of changes in organisations because it can buffer the negative effects of change. It can influence the way a change is perceived and research shows that it is precisely this subjective experience of change that has the greatest impact on the results of a change. At the same time, results of studies regarding subjective change explain and support what we know from research on change in organisations; specifically the importance of the availability of information, sense of control, expectation of personal gains, and the roles of leaders—those who implement change.

Coaching influences people’s thinking and emotions, and thus it has the potential to support constructive, positive thinking by emphasising the opportunity for development associated with change. Managers are particularly worth supporting, especially those responsible for planning and implementing change, because they are under the most pressure—both experiencing the change and introducing the change—and the reactions of employees as well as the very fate of the change itself depends on their attitudes and the solutions they propose.

## Concluding Remarks

In the contemporary world organisations change frequently and quickly. However, most often changes do not bring expected results. For a long time the role of the recipients of the changes, the “human factor,” employees, were underestimated. Many authors suggested resistance of the recipients of change is the main reason why changes often fail. Therefore, we aimed to investigate change from a broader perspective as a general individual reaction to change and change in the organisational context. We also tried to describe coaching as a potential method which helps with implementing changes in organisation.

Our first research question: *how people experience change in their life and what influences attitude to change* referred to a broad perspective on change. It was shown that every change, negative or positive, can be related to negative emotional consequences, because it is a demanding self-regulatory task for individuals, also it often unsettles consistency of self-image. However, subjective perception of the change is more important to long lasting consequences of the change than its objective characteristics. Important factors that can influence attitude to change are positive beliefs about change, sense of control and meaningful relationships with others.

Second question: *what factors are crucial in individual resistance to change implemented by an organisation* referred to change in organisational context. As in the broad perspective of change, subjective perception of change by employees seems to be decisive in effective implementation of changes. Resistance to change is affective, cognitive and behavioural phenomena based on individual characteristics and former experiences. Emotions are the most crucial factor, which trigger change in an individual. Thus, emotions of the recipients of change should be always taken into account.

Third question: *how can coaching help in implementing change in organisation* referred to practical implications of above considerations. Research on effects of coaching during organisational change are scarce. However, general research on coaching and practical experience suggests that it can be a valuable method in minimising negative effects of changes. Coaching can be implemented at different levels of organisations: top management, teams, individual employees and at different stages of change: planning, communicating and implementing. It can also be used to deal with emotions related to change. Coaching itself is also a method that emphasises subjective perspective. These arguments suggest that coaching can help effectively with change in organisation.

It is necessary to conduct research on subjective change and its influence on change in organisations as well as to encourage organisations to use coaching and to verify its influence by taking part in research projects.

## Data Availability Statement

The original contributions presented in the study are included in the article/supplementary material, further inquiries can be directed to the corresponding author.

## Author Contributions

All authors listed have made a substantial, direct, and intellectual contribution to the work, and approved it for publication.

## Conflict of Interest

The authors declare that the research was conducted in the absence of any commercial or financial relationships that could be construed as a potential conflict of interest.

## Publisher’s Note

All claims expressed in this article are solely those of the authors and do not necessarily represent those of their affiliated organizations, or those of the publisher, the editors and the reviewers. Any product that may be evaluated in this article, or claim that may be made by its manufacturer, is not guaranteed or endorsed by the publisher.

## Footnotes

1 When system acts in accordance to specific rules it maintains balance (homeostasis), which is a condition for its efficiency; if a rule cannot be realised, the system loses balance and has difficulty functioning, which is why the system seeks to regain homeostasis (and, in doing so, the functional fitness of the system) using two feedback mechanisms:

–negative feedback, i.e., a process which reinstates balance through returning to previous state, the “old rule”;
–positive feedback, i.e., a process which reinstates balance through changing the system, finding new rules.
